# Glioblastoma in NF1: A Unique Entity—A Literature Review Focusing on Surgical Implication and Our Experience

**DOI:** 10.3390/curroncol32040242

**Published:** 2025-04-21

**Authors:** Elisa Garbin, Lorenzo Nicolè, Salima Magrini, Yuri Ceccaroni, Luca Denaro, Luca Basaldella, Marta Rossetto

**Affiliations:** 1 Academic Neurosurgery, Department of Neurosciences, University of Padova, 35128 Padova, PD, Italy; luca.denaro@unipd.it; 2 Unit of Pathology, Angel Hospital, 30174 Mestre, VE, Italy; lorenzo.nicole@aulss3.veneto.it; 3 Unit of Neurosurgery, Department of Neurosciences, Angel Hospital, 30174 Mestre, VE, Italy; salima.magrini@aulss3.veneto.it (S.M.); yuri.ceccaroni@aulss3.veneto.it (Y.C.); luca.basaldella@aulss3.veneto.it (L.B.)

**Keywords:** glioblastoma, high-grade glioma, neurofibromatosis type one, recurrent glioblastoma, long-term survival

## Abstract

Glioblastoma in patients affected by NF1 germline mutation (NF1-associated GBM) represents a unique heterogeneous clinical and pathological entity. We have reviewed the few cases reported in the literature and they seem to have a better response to standard therapy and overall survival than GBM in the non-NF1 population. We present two cases of long-survival NF1 patients with GBM. Case 1 was a 38-year-old woman with cerebellar GBM who underwent surgical asportation and the Stupp protocol many times with an overall survival of 117 months. Case 2 was a 47-year-old woman with GBM in the eloquent area of the right frontal lobe; she underwent surgical asportation and the Stupp protocol with an overall survival of 25 months. The data analysis demonstrates that NF1-associated GBM patients could be considered long-term survivors.

## 1. Introduction

Glioblastoma (GBM) is an aggressive glial tumor, characterized by poor prognosis and one of the worst five-year survival rates in all malignancies [1,2]. The incidence is 2–3 per 100,000 persons, and patients who live more than three years are considered long-term survivors (LTS) [2,3]. In recent years, despite advances and multimodal therapies, only 5% of GBM patients achieve 5-year survival, and this percentage is not increasing. To date, from a histological point of view, LTS-GBMs are not different from STS-GBMs (short-term survivors). Despite a lot of effort in the molecular field, in a recent multicenter retrospective study, no new morphological or routine molecular features can characterize or distinguish LTS from STS [4]. The GBM standard of care is based on maximal safe resection (MSR) followed by chemotherapy and radiotherapy, according to the Stupp protocol. Nevertheless, treatment is not curative and GBMs inevitably recur. Following recurrence, only a minor proportion of patients could be eligible to receive further surgery [5,6].

In the current literature, no standards of care are established for recurrent GBM; therefore, treatment is based on clinical practice and not on recognized guidelines [5,6,7]. Several prognostic factors need to be taken into consideration to select the second-line treatments, such as tumor size and location, performance status, comorbidity, and the time frame from previous radiation. Recurrent GBM surgery should be carefully evaluated and considered in selected patients even more than first surgery: first, because adjuvant therapies are lacking so it is important to achieve a gross total resection (GTR); on the other hand, because recurrent-GBM patients can have higher risks of post-operative complications.

The inherited neurocutaneous disease neurofibromatosis type one (NF1) increases the chance of developing central nervous system tumors and NF1 patients are at higher risk of malignant high-grade gliomas [1,3,8,9].

GBM arising in the setting of NF1 germline mutation (NF1-associated GBM) is a unique heterogeneous entity, occurring from childhood through adulthood; according to different genetic patterns, these tumors follow an indolent or aggressive clinical course [1,3]. Nevertheless, several reports agree that patients with NF1-associated GBM have prolonged survival than non-NF1-associated GBM patients [2,8,10].

We have reviewed the literature about the pathology [8], and we present our experience regarding two cases of patients with NF1-associated GBM. The main aim of this literature systematic review is to evaluate the current knowledge on the treatment and survival of patients with NF1-associated GBM; we also analyzed genetic data, which could be decisive in defining a multimodal, repeatable, and more effective treatment addressed to this subgroup of GBMs.

## 2. Materials and Methods

### 2.1. Research Strategy

We conducted an online systematic literature review according to the Preferred Reporting Items for Systematic Reviews and Meta-Analyses (PRISMA) recommendations [11]. We considered only articles available in the English language. Articles that were published between the databases’ date of inception and 31 December 2023 were considered. The electronic database PubMed and Google Scholar were examined. The combination of MeSH terms and free-text-keywords used in the search process was “glioblastoma”, “high-grade glioma”, “neurofibromatosis type one”, “recurrent glioblastoma”, “long-term survival”, and “recurrence in glioblastoma”. All the included articles were collected and analyzed independently by the authors and cross-checked for accuracy.

### 2.2. Selection Criteria

Studies were considered eligible according to the following inclusion criteria: (I) confirmed anatomo-pathological diagnosis of GBM in patients with a germline NF1 mutation; (II) patients more than 14 years old; and (III) studies written in the English language. The exclusion criteria were as follows: (i) lack of relevant data; (ii) studies on the pediatric population; (iii) non-original studies (i.e., reviews, recommendations, letters, editorials, conference papers, and book chapters); and (iv) studies not written in English.

### 2.3. Data Extraction, Quality Assessment, and Data Analysis

Extracted data from each article were collected in an electronic database including the first author, year of publication, age and sex, tumor localization, treatment, recurrence, treatments after recurrence, survival, pathological diagnosis, and molecular diagnosis (GFAP, S100, p35 mutation, Ki67% expression, EGFR amplification, MGMT methylation, IDH1 mutation, BRAF-V600E mutation, TERT promoter mutation, and ATRX mutation). Each relevant article was carefully evaluated to collect information; two reviewers independently evaluated the papers, and any disagreement was resolved by discussion. For each parameter, we calculated the mean and standard deviation.

## 3. Cases

### 3.1. Case One

We present the case of a 38-year-old woman with a germline mutation of the NF1 gene who developed cerebellar glioblastoma. She presented to the emergency department complaining of a progressive headache. Neuroimaging with a brain CT scan showed a large heterogeneous mass lesion in the right cerebellar hemisphere measuring approximately 5.0 × 4.0 cm associated with extensive surrounding edema. The brain CT scan was indicative of initial acute hydrocephalus caused by a posterior cranial fossa lesion, distorting and obstructing the fourth ventricle. A brain MRI confirmed a gadolinium-enhanced lesion with internal areas of cysts and necrosis. The patient underwent microscopic gross total resection, and immunohistochemical results of the intracranial specimen showed WHO grade IV glioblastoma. Histological examination confirmed an infiltrative glial lesion: immunoreaction positive for GFAP and for oligodentrocyte transcription factor (OLIG2) in tumor cells. Histological examination also showed increased cellularity, moderate atypia, few mitotic figures (four mitoses for 10 HPF), no necrosis, and microvascular proliferation was observed. Immunoreaction for isocitrate dehydrogenase (IDH1) was negative, nuclear ATRX resulted in loss, and p53 showed a typical wildtype staining pattern. Due to the young age of the patient, a molecular test for IDH1 and IDH2 status was also performed in order to investigate non-canonical *IDH* mutations. An IDH molecular test result was negative for both genes. No mutations of the telomerase reverse transcriptase (TERT) gene promoter were identified. The promoter of the methylguanine methyltransferase (*MGMT*) gene resulted in methylated. A post-surgery brain MRI showed no residual disease. The patient’s post-operative course was uneventful, and she received standard concomitant chemoradiotherapy followed by adjuvant chemotherapy with temozolomide (the Stupp protocol). She remained asymptomatic for the next 6 years and then presented once again complaining of a headache and vertigo. The first recurrence of a tumor was then detected at a 6-year follow-up brain MRI; neuroimaging showed a recurrent cystic lesion with heterogeneous enhancement of the previous surgical field margin. The patient underwent tumor resection once again. Concurrent chemoradiotherapy was possible considering the time lapse from the previous irradiation. The immunohistochemical results of the intracranial specimen showed no change in the histology. She had a regular brain follow-up MRI; a second tumor recurrence was detected after another 3 years. Since the patient had a good clinical condition and KPS score (Karnofsky score), she was treated once again with surgical resection followed by chemoradiotherapy. The histology and the molecular features were confirmed to be the same. At the 7-month follow-up, there was clinical and radiological progression involving the brainstem, and was thus inoperable; she subsequently died.

### 3.2. Case Two

The second case was a 47-year-old woman with NF1 who presented with frontal GBM. She underwent a brain MRI during an NF1 follow-up, and she was found to have a large mass lesion in the right frontal lobe with contrast enhancement associated with surrounding edema. The neurological examination was normal. Gross total resection was achieved, and concurrent chemotherapy and radiotherapy were administered (the Stupp protocol). Histological examination confirmed an infiltrative glial lesion (immunoreaction positive for GFAP and negative for OLIG2 in tumor cells), with high cellularity, severe atypia with diffuse pleomorphism, increased mitotic figures (10 mitoses for 10 HPF), diffuse necrosis, and microvascular proliferation was also observed. Both immunoreactions for IDH1 and ATRX resulted in negative, while p53 resulted in a typical wildtype staining pattern. An IDH molecular test resulted in negative for both IDH1 and IDH2 genes, and also for non-canonical IDH mutations.

No mutations of the TERT gene promoter were identified. The promoter of the MGMT gene resulted in methylation. A post-operative brain MRI showed no residual disease.

Unfortunately, after 25 months, she presented with a headache and left paresis; a brain MRI revealed a recurrence of the tumor in an eloquent area extending toward the internal capsule, so second surgery was excluded. She was then treated conservatively with chemotherapy, and she died after 4 months.

## 4. Results

### 4.1. Search Result and Quality Assessment

**Figure 1:**
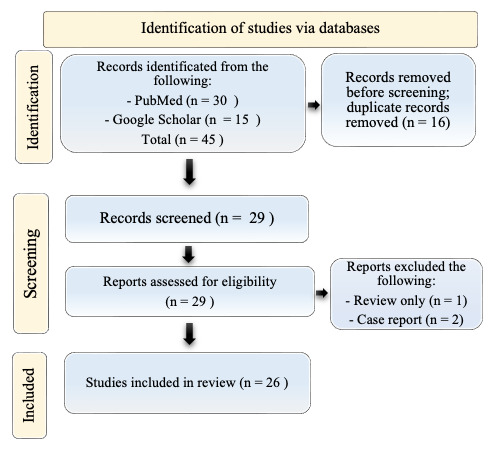
PRISMA diagram resembling the electronic database search and inclusion/exclusion process of the review. Legend: the date of the last search was 31 December 2023. The image is not reprinted/has not been previously published.

A total of 29 titles were collected from our literature search. After the exclusion of 3 records due to coherence with the inclusion/exclusion criteria, 26 articles relevant to the topic were examined and included in the review. A detailed flowchart of the search process is shown in Figure 1.

### 4.2. Included Studies’ Characteristics

To our knowledge, there are little more than thirty cases of NF1-associated GBM reported in the literature, excepting the two present cases. These studies were published between 1997 and 2023. In Table 1, we reported a summary of clinical and therapeutic features of patients with NF1 diagnosed with glioblastoma. The data on tumor molecular findings are reported in Table 2.

### 4.3. Demographic Data of NF1-Associated High-Grade Glioma

The patients’ ages ranged from 14 to 63 years, with a mean age at diagnosis of 34.2 (±13.6) years and male gender prevalence (72% male, 28% female). The most common location was the frontal lobe (10/32; 31%) followed by the cerebellum (9/32; 28%); other locations were less common: the thalamus or midbrain (4/32), occipital lobe (2/32), parietal lobe (2/32), temporal lobe (3/32), insula (1/32), and peri-trigonal region (1/32). Our cases are in line with the literature concerning location, while the patients’ age at diagnosis was a little higher than in the literature studies. Our cases diverge from the literature on gender prevalence [6].

### 4.4. Treatment and Recurrence of NF1-Associated High-Grade Glioma

The majority of patients underwent gross total or subtotal resection followed by radiotherapy and chemotherapy. Three of thirty-two patients were excluded from the data analysis about treatment or survival because they received palliative treatment or refused any treatment [12,14,26]. A total of 3/29 patients underwent a biopsy followed by radiotherapy and chemotherapy [26,27]. A total of 2/29 patients underwent only surgical resection [18,22]. A total of 16/29 had a record of recurrence, and the mean time of the recurrence after diagnosis was 24 (±31.5 SD) months [8,13,15,16,17,18,22,23,29,31]. A total of 11/29 did not have recurrence at the time of the last follow-up, with a mean progression-free survival of 52.7 SD months [8,18,19,20,21,23,24,25,30,32,33].

### 4.5. Treatment After Recurrence and Overall Survival in NF1-Associated High-Grade Glioma

The mean time of survival was 44.6 (±44.1 SD) months and 8/28 (28.6%) patients were alive at the 5-year follow-up. The majority of patients with recurrence or progression have had second treatments. The data are extremely variable and lacking; however, many patients underwent repeat surgical resection, chemotherapy, and radiotherapy in various combinations [8,18,23,28,29,31,33]. We think these data are significant because unfortunately it is known that patients with non-NF1-associated GBMs have a survival rate of only 5.8% at 5 years post-diagnosis [1,6]. Our cases revealed an extremely long survival of 117 months (Case 1) and 29 months (Case 2).

## 5. Discussion

Gliomas arising in the setting of NF1 are driven by biallelic NF1 inactivation: tumors develop following somatic inactivation of the remaining wildtype allele through either the loss of heterozygosity (LOH) or a second tumor-acquired mutation [1,3,35]. The data collected showed that the mean age at the diagnosis of patients with NF1-associated high-grade is much younger than that of patients with sporadic glioblastoma (mean age 34 vs. 62 years in GBM-IDHwt-44 years in high-grade astrocytoma-IDHmut) [1,6]. The scientific literature offers limited data on GBM in patients with NF1, but some reports agree that NF1 could have a role in increasing the median survival in a percentage of patients with GBM.

An interesting point to address is the difference in survival outcomes between sporadic glioblastomas and NF1-associated GBMs [36]. As shown in Table 1, the mean time of survival was 44.6 (±44.1 SD) months in NF1-associated GBMs compared to 15 months in sporadic GBM [2]. Sporadic GBMs are typically associated with more aggressive behavior and poorer prognosis [1]. Conversely, NF1-associated GBM often exhibits distinct molecular characteristics, such as increased immune activation and a lower mutational burden, which may account for the improved survival outcomes reported in the literature [37]. Furthermore, earlier diagnosis, younger age in NF1 patients, and differences in treatment responsiveness likely play a role. These discrepancies warrant further investigation, as they could provide valuable insights into tailored therapeutic approaches for these distinct subgroups [8,33,38].

### 5.1. Pathology of NF1-Associated High-Grade Glioma

NF1-associated GBMs are a heterogeneous group of neoplasms driven by biallelic *NF1* inactivation [1]. The first group represents low-grade gliomas that commonly occur in young people, sharing morphological features with pilocytic astrocytoma, and affects preferentially the optic pathway, cerebellum, and thalamus. Recently, a study comprising 47 NF1-associated gliomas showed that those patients with low-grade gliomas, independently from morphological presentation (conventional pilocytic astrocytoma, diffuse astrocytoma, or ganglioglioma), presented with tumors in a single specific epigenetic cluster [1]. Typically NF1-associated low-grade gliomas present an indolent clinical course. On the other hand, NF1-associated high-grade gliomas represent an epigenetically heterogeneous group sharing typical high-grade histological aspects [38]. Some of them align in a cluster of high-grade tumors with piloid features, while most of them share epigenetic profiles with various types of IDH-wildtype glioblastoma. Piloid features in a setting of high-grade glioma may harbor *ATRX* mutation and *CDKN2A* deletion. In contrast, high-grade tumors without piloid features, despite harboring *CDKN2A (*cyclin-dependent kinase inhibitor 2A*)* deletion, rarely show *ATRX* mutation. TERT promoter mutations or *EGFR* amplification have not yet been described in the setting of NF1-associated gliomas, while alteration as a gain of chromosome 7 and loss of 10 were rarely observed. High-grade glioma arising in NF1 occurs primarily in adults presenting a worse outcome compared to NF1-low-grade gliomas. However, those high-grade tumors with piloid features seem to present an outcome intermediate between IDH-mutant astrocytoma and sporadic IDH-wildtype glioblastomas [39,40].

**Figure 2:**
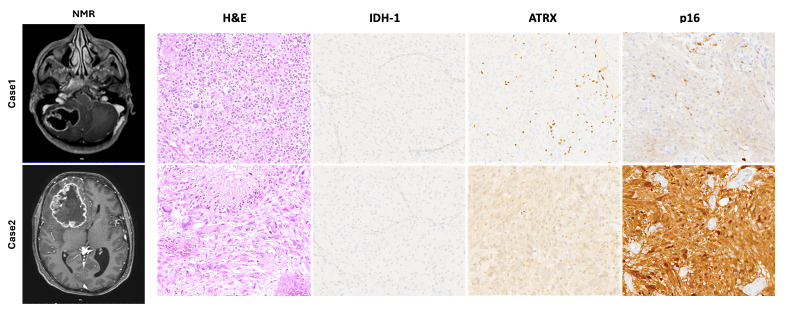
Representative pre-operative NMR images showing the anatomical location of the lesion in both cases described. Hematoxylin and eosin (H&E) stained slides showed a typical high-grade glial lesion with increased cellularity, severe atypia, necrosis, and a high mitotic rate. Immunohistochemical stains, as described in the main text, confirmed the absence of an *IDH-1* mutation and the presence of an *ATRX* mutation, which represent a typical molecular profile of glioblastomas arising in a setting of NF1. Only Case 1 showed a loss of p16 suggesting homozygous deletion of *CDKN2A.* Microphotographic images were captured at an original magnification of 10×. The images are not reprinted/have not been previously published.

The cases presented here (Figure 2) clearly represent the two high-grade glioma groups described. Case 1 showed piloid morphology with *ATRX* mutation, loss of p16 (*CDK2NA* deletion), and a longer survival time compared with Case 2, which showed morphology and a molecular status similar to sporadic IDH-wildtype glioblastomas (nuclear ATRX and p16 retained). Moreover, previous studies have shown that the loss of ATRX increases sensitivity to DNA-damaging agents [39], suggesting that ATRX mutations may represent a crucial point of therapeutic intervention for high-grade NF1 gliomas and LGm6 sporadic gliomas [37,40,41].

### 5.2. Treatment and Recurrence in NF1-Associated High-Grade Glioma

As reported by Miele et al. in their multicentric retrospective study on IDH-wt-GBM, they did not find significant molecular differences between LTS and STS. The only variable that had an impact on LTS was KPS; indeed, the higher KPS showed a trend with longer survival. The younger age at diagnosis in patients with NF1-associated high-grade gliomas could affect the better KPS and partially explain the longer survival [4]. It is already known that KPS plays a significant role in predicting survival; Brotos et al. reported significant increases in OS, and it seems to be obtained with the maximization of both the extent of resection (EOR) and KPS [42].

While the Stupp protocol is almost standardized, the treatment at recurrence still lacks consensus. Brotos et al.’s systematic review of repeat resections (RRs) in recurrent non-NF1-associated GBM (rGBM) found a survival benefit for RR at first recurrence, whereas no survival benefits were found on the second (or more) recurrence surgery. The median survival time ranges from 6 to 17 months, with total or subtotal resection (EOR > 80%) being associated with longer survival [42].

A recent retrospective study of the RANO resect group investigated the prognostic role of surgery for recurrent non-NF1-associated GBM. They found that patients without residual contrast-enhancement tumors experience substantially longer survival following re-resection. Moreover, they created the “RANO classification system” based on the presence of a residual tumor on an MRI as a prognostic stratification tool. They recommend surgery for recurrence if a residual contrast-enhancement tumor is expected to be ≤1 cm^3^ because this is correlated with a favorable outcome after re-resection. In addition, they suggest radio-chemotherapy may consolidate the beneficial effects of re-resection [43].

In 2017, ASCO endorsed the ASTRO guidelines on radiation therapy for glioblastoma; according to an ASCO review, “In younger patients with good performance status, focal reirradiation (e.g., stereotactic radiosurgery, hypofractionated stereotactic radiotherapy, brachytherapy) for recurrent glioblastoma may improve outcomes compared with supportive care or systemic therapy alone (LQE). Tumor size and location should be taken into account when deciding whether reirradiation would be safe (LQE; weak recommendation)” [38].

However, no evidence supports reirradiation in GBM because the majority of these studies have been retrospective; they tended to select from smaller volumes of disease, and no standard volume or cross-sectional diameter has been determined as optimal for reirradiation [44]. On the other hand, Minniti et al. recently presented a clinical overview of the current status and advances of reirradiation in the setting of recurrent or progressive GBM after standard treatment. They found that reirradiation is an efficient and safe treatment in the management of recurrent GBM. They also emphasized the need for appropriate patient selection (young patients with good performance status, especially after a long period from prior radiation) [7,38,44,45].

According to the authors and data from the literature review, NF1-associated GBMs, due to the characteristic younger age and the longer mean time of recurrence, are more eligible and tolerant to new irradiation.

## 6. Limitations

Our analysis has some important limitations. First, only a few cases are included in the studies because of the rarity of the pathology. So, it is challenging to derive the survival rate or best treatment from the few reported cases. Moreover, there is a lack of uniformity in each center, and the treatment decision is based on a single-case experience. Another significant limitation is the risk of misdiagnosis of GBM across the studies.

## 7. Conclusions

Glioblastoma in patients affected by NF1 germline mutation delineates a unique entity. The mean age at diagnosis in these patients is effectively younger than in non-NF1-associated GBM. Moreover, progression-free survival and overall survival are noticeably longer [1,5,8,29,32]. This peculiar trend should be kept in mind, especially in recurrent NF1-associated GBM. We think surgical indication in this subgroup of patients can be considered on recurrence even multiple times, according to PFK. We believe more studies and larger series are necessary to better understand the causes underpinning this unique subset of GBM, their prognosis, and therapy implications in order to achieve proper patient management.

## Figures and Tables

**Table 1:** Summary of clinical and therapeutic features of patients with NF1 diagnosed with glioblastoma reported from 1997 to 2023.

	Authors and Year	Ref	Age (Years) and Sex	Location	Treatments	Recurrence or Progression (Months)	Treatment After Recurrence	Survival (Months)
1	Miaux et al., 1997	[12]	32/F	Occipital	Biopsy	NA	NA	NA
2	Miyata et al., 2005	[13]	30/F	Right Frontal	SR + RT + CHT	10	NA	10 (recurrence then lost)
3	Mehta et al., 2008	[14]	63/M	Parietal	Biopsy (refused any treatment)	2	-	2 (died)
4	Hakan et al., 2008	[15]	28/F	Frontal	SR + RT + CHT	41	-	41 (died)
5	Broekman et al., 2009	[16]	28/F	Right Cerebellar	SR + RT + CHT	6	-	12 (died)
6	Taraszewska et al., 2012	[17]	29/F	Cerebellar Hemisphere	SR	0	-	0 (died)
7	Theeler et al., 2014	[18]	59/M	Right Temporal	SR + RT + CHT	24		104 (last follow-up: disease control)
8	Theeler et al., 2014	[18]	25/M	Thalamus	RT + CHT	2	CHT	14 (died)
9	Theeler et al., 2014	[18]	32/M	Cerebellar Hemisphere	RT + CHT	3	CHT	73 (died)
10	Jeong et al., 2014	[19]	32/M	Right Frontal	SR+ RT + CHT	-	-	9 (last follow-up: disease control)
11	Varghese et al., 2015	[20]	60/M	Right Frontal	SR + RT + CHT	NA	NA	NA
12	Belsuzarri et al., 2015	[21]	58/M	Left Parietal	SR + RT + CHT	-	-	10 (last follow-up: disease control)
13	Ameratunga et al., 2016	[22]	24/M	Left Cerebellar	SR + RT + CHT	2 (brainstem extension)	SR + CHT	24 (last follow-up: disease control)
14	Shibahara et al., 2018	[23]	52/M	Right Occipital	SR + RT + CHT	49	-	49 (last follow-up: died)
15	Shibahara et al., 2018	[23]	34/M	Right Frontal	SR + RT + CHT	-	-	106 (last follow-up: disease control)
16	Shibahara et al., 2018	[23]	28/M	Left Insula	SR + RT + CHT	-	SR + CHT + RT	60 (last follow-up: disease control)
17	Shibahara et al., 2018	[23]	53/M	Left Frontal	SR + RT + CHT	-	-	87 (last follow-up: disease control)
18	Singla et al., 2018	[24]	25/M	Right Frontal	SR + RT	-	-	36 (last follow-up: disease control)
19	Fortunato et al., 2018	[25]	23/M	Brainstem	SR + RT + CHT	-	-	1 (last follow-up, then lost)
20	Picart et al., 2018	[26]	38/F	Cerebellar Hemisphere	SR	NA	NA	NA
21	Picart et al., 2018	[26]	41/M	Cerebellar Hemisphere	SR + RT	2	NA	6 (died)
22	Picart et al., 2018	[26]	28/F	Vermis	Biopsy + RT + CH	25	NA	32 (last follow-up)
23	Wong et al., 2019	[27]	27/M	Multiple (bitalamic)	Biopsy + RT + CHT	-	-	39 (last follow-up: disease control)
24	Narasimhaiah et al., 2019	[28]	21/F	Right Frontal–Parietal	SR + RT + CHT	-	-	32 (last follow-up: disease control)
25	Narasimhaiah et al., 2019	[28]	16/M	Right Peri-trigonal	SR (subtotal)	120	SR	120 (last follow-up)
26	Flower and Gallo., 2019	[29]	23/M	Cerebellar	SR + RT + CHT	17	CHT	18 (died)
27	Derakhshan et al., 2019	[30]	33/M	Cerebellar	SR (subtotal) + RT + CHT	-	-	19 (last follow-up: disease control)
28	Awada et al., 2020	[31]	19/M	Brainstem: mesencephalic	RT + CHT	6	CHT + molecular targeted therapy (third-line and fourth-line treatment)	36 (last follow-up: disease control)
29	Cai et al., 2021	[32]	51/F	Right Temporal	SR + CHT	-	-	13 (last follow-up: disease control)
30	Basindwah et al., 2022	[8]	27/M	Left Fronto–Parietal	SR + RT +CHT	48	SR (debulking) + RT + CHT + molecular targeted therapy	121(last follow-up: further progression, patients refused treatment)
31	Al-romaihi et al., 2023	[33]	14/M	Left Frontal	SR + RT +CHT	-	-	168 (last follow-up: disease control)
32	M.A. Alla et al., 2023	[34]	43/M	Left Temporal	Biopsy + RT + CHT	5	-	10 (died)
33	Present case 1		36/F	Right Cerebellar	SR + RT+ CHT	72	SR + RT + CHT	117 (last follow-up: died)
34	Present case 2		47/F	Right Frontal	SR + RT + CHT	25	CH	29 (last follow-up)

NA = not available; RT = radiotherapy; SR = surgery resection; CHT = chemotherapy.

**Table 2:** Summary of pathological findings of patients with NF1 diagnosed with glioblastoma reported from 1997 to 2023.

	Author/Year	Ref	Pathological Findings	GFAP	S100	Ki67%	EGFR Amplification	MGMT Methylation	P53	IDH1 Mutation	BRAF V600E Mutation	TERT Promoter Mutation	ATRX Mutation
1	Miaux et al.,1997	[12]	Examination shows pathological characteristics of glioblastoma.	NA	NA	NA	NA	NA	NA	NA	NA	NA	NA
2	Miyata et al.,2005	[13]	“In the periphery of tumor, anaplastic cells with pleomorphic nuclei proliferate diffusely with marked vascular proliferation and ischemic necrosis, which was considered as features of glioblastoma. The majority of the tumor consisted of compact dark cells and large gemistocyte-like eosinophilic cells forming prominent tubular or glandular structures supported by abundant basophilic myxomatous stroma.”	+	NA	58%	NA	NA	-	NA	NA	NA	NA
3	Mehta et al.,2008	[14]	Examination shows pathological characteristics of glioblastoma.	NA	NA	NA	NA	NA	NA	NA	NA	NA	NA
4	Hakan et al.,2008	[15]	Examination shows pathological characteristics of glioblastoma.	NA	NA	NA	NA	NA	NA	NA	NA	NA	NA
5	Broekman et al., 2009	[16]	“Mitotically active pleomorphic astrocytoma with pathologic vascular proliferation that was classified as glioblastoma multiforme.”	+	-	15%	NA	NA	30%	NA	NA	NA	NA
6	Taraszewska et al., 2012	[17]	“Pleomorphic giant and small tumor cells and focal necrosis with pseudopalisading pattern. Coagulative necrosis surrounded by giant and small tumor cells.”	+	NA	NA	NA	NA	+	NA	NA	NA	NA
7	Theeler et al., 2014	[18]	Examination shows pathological characteristics of glioblastoma.	NA	NA	NA	NA	NA	NA	NA	NA	NA	NA
8	Theeler et al., 2014	[18]	Examination shows pathological characteristics of glioblastoma.	NA	NA	NA	NA	NA	NA	NA	NA	NA	NA
9	Theeler et al., 2014	[18]	Examination shows pathological characteristics of glioblastoma.	NA	NA	NA	NA	NA	NA	-	NA	NA	NA
10	Jeong et al.,2014	[19]	Examination shows pathological characteristics of glioblastoma.	+	NA	10%	-	-	NA	NA	NA	NA	NA
11	Varghese et al.,2015	[20]	Examination shows pathological characteristics of glioblastoma.	NA	NA	NA	NA	NA	NA	NA	NA	NA	NA
12	Belsuzarri et al., 2015	[21]	“Giant cell and an increased number of giant cells and pleomorphic zone with more aggressive component.”	NA	NA	NA	NA	NA	NA	NA	NA	NA	NA
13	Ameratunga et al., 2016	[22]	Pregress optic glioma.	NA	NA	NA	NA	NA	NA	NA	NA	NA	NA
14	Shibahara et al., 2018	[23]	“Most of the tumor cells possessed large round-to-oval eosinophils with bizarre multiple nuclei throughout the cytoplasm. Many tumor cells were similar to epithelioid glioblastoma cells, showing eccentrically located nuclei with round eosinophilic intracytoplasmic inclusions. Widespread necrotic areas and small foci of microvascular proliferation (MVP) were present at the center of the tumor tissue.”	NA	NA	20%	-	low	10%	-	-	-	-
15	Shibahara et al., 2018	[23]	“The tumor was composed of large polygonal-shaped cells with bizarre nuclei and elongated spindle-shaped cells. Some giant neoplastic cells showed xanthomatous change or many microvacuoles in the cytoplasm resembling PXA. MVP and necrotic foci with nuclear palisading were confirmed in the tumor tissue.”	NA	NA	38%	-	low	20%	-	-	-	-
16	Shibahara et al., 2018	[23]	“The tumor cells were mainly round-to-polygonal in shape with large areas of cytoplasm and bizarre hyperchromatic nuclei. Multinucleated giant cells were also occasionally encountered. The bizarre giant cells and mid-sized round tumor cells showed distinctive xanthomatous change resembling PXA. Proliferation of elongated spindle-shaped tumor cells in fascicular or storiform patterns was also evident.”	NA	NA	40%	-	high	10%	-	-	-	-
17	Shibahara et al., 2018	[23]	“The tumor was composed of round-to-polygonal giant multinucleated cells and spindle-shaped cells, densely proliferating in a storiform pattern. The cytoplasm of the giant cells showed xanthomatous change resembling PXA. Abnormal mitotic figures sporadically occurred. Though no MVP was detected, the presence of a few minute necrotic foci ensured the histological diagnosis of glioblastoma.”	NA	NA	20%	-	high	10%	-	-	-	-
18	Singla et al., 2018	[24]	“Pregress pleomorphic xanhoastrocytomaGBM highly cellular with areas of necrosis and moderate nuclear pleomorphism with frequent mitotic activity Ki-67 immunostain shows a high proliferative index.”	NA	NA	high	NA	NA	NA	NA	NA	NA	NA
19	Fortunato et al., 2018	[25]	Examination shows pathological characteristics of glioblastoma (H3K27 negative).	+	-	30%	NA	NA	NA	-	NA	NA	+
20	Wong et al.,2019	[27]	Examination shows pathological characteristics of glioblastoma. KMT2B mutation.	NA	NA	NA	NA	NA	NA	+	NA	NA	NA
21	Picart et al., 2018	[26]	Examination shows pathological characteristics of glioblastoma.	NA	NA	NA	NA	+	NA	-	NA	-	NA
22	Picart et al., 2018	[26]	Examination shows pathological characteristics of glioblastoma.	NA	NA	NA	NA	-	NA	-	NA	-	NA
23	Picart et al., 2018	[26]	Examination shows pathological characteristics of glioblastoma.	NA	NA	NA	NA	+	NA	-	NA	-	NA
24	Narasimhaiah et al., 2019	[28]	“High-grade astrocytoma with numerous giant cells; pleomorphic and mitotically active nuclei with admixed tumor giant cells and bizarre nuclear forms. Also present were foci of geographic and pseudopalisading necrosis.”	+	+	30%	NA	NA	+	-	NA	NA	-
25	Narasimhaiah et al., 2019	[28]	“Glioblastoma with foci of pseudopalisading necrosis and vascular proliferation.”	+	+	30%	NA	NA	+	-	NA	NA	+
26	Flower and Gallo., 2019	[29]	“Glioblastoma with PNET differentiation and MGMT methylation.”	NA	NA	NA	NA	+	NA	NA	NA	NA	NA
27	Derakhshan et al., 2019	[30]	“Hypercellularity with neurofibrillary background and areas of microcytic pattern with vascular proliferation and foci of necrosis in favor of GBM. Tissue cells showed pleomorphism, atypia, and hyperchromasia. Mitosis was 1–2/Hpf. (CD34– ; Oligo-2+).”	+	+	5%	NA	NA	-	-	NA	NA	NA
28	Awada et al., 2020	[31]	Examination shows pathological characteristics of glioblastoma.	+	NA	5–10%	NA	NA	+	NA	NA	NA	+
29	Cai et al.,2021	[32]	Examination shows pathological characteristics of glioblastoma.	+	+	30%	NA	+	80%	-	-	-	NA
30	Basindwah et al., 2022	[8]	“Anaplastic or giant cells with marked nuclear atypia and pleomorphism and sheets of smaller round-to-oval cells. The cytoplasm amount was variable, from scant to abundant, and many cells were exhibiting a minigemistocyte appearance. Microvascular proliferation and areas of necrosis were evident. 1p/19q codeletion.”	+	NA	20%	NA	NA	NA	-	NA	NA	NA
31	Al-romaihi et al., 2023	[33]	“Shows hypercellular tumors with large areas of geographic necrosis (pinkish and bluish areas); highly atypical pleomorphic tumor cells, including multinucleated forms with many atypical mitotic figures. Positive for vimentin, GFAP (patchy positive), S100 (weak positive), overexpression of P53, and CD68.“	+	+	70%	-	NA	NA	-	NA	NA	NA
32	M.A. Alla et al., 2023	[34]	“Diffuse high-grade glioma, showing strongly atypical large tumor cells. Multinucleated giant cells are also noted.”	+	NA	NA	NA	NA	-	-	NA	NA	-
33	Present case 1		Infiltrative glial lesion (immunoreaction positive for GFAP and OLIG2 in tumor cells), with increased cellularity, moderate atypia, few mitotic figures (4 mitosis for 10 HPF), no necrosis, and microvascular proliferation was observed.	+	NA	NA	NA	+	-	-	NA	-	-
34	Present case 2		Infiltrative glial lesion (immunoreaction positive for GFAP and negative for OLIG2 in tumor cells), with high cellularity, severe atypia with diffuse pleomorphism, increased mitotic figures (10 mitosis for 10 HPF), diffuse necrosis, and microvascular proliferation was observed.	+	NA	NA	NA	+	-	-	NA	-	-

+: mutated; -: not mutated; NA: not available; GFAP: glial fibrillary acidic protein; S100: S100 protein; p53: p53 protein; EGFR: epidermal growth factor receptor; MGMT: methylguanine-DNA-methyltransferase; IDH: isocitrate dehydrogenase; TERT: telomerase reverse transcriptase; ATRX: ATRX chromatin remodeler.

## Data Availability

All data generated or analyzed during this study are included in the published paper.
